# Microsaccades reflect attention shifts: a mini review of 20 years of microsaccade research

**DOI:** 10.3389/fpsyg.2024.1364939

**Published:** 2024-02-19

**Authors:** Quan Gu, Qikai Zhang, Yueming Han, Pengfei Li, Zaifeng Gao, Mowei Shen

**Affiliations:** ^1^Yongjiang Laboratory, Ningbo, China; ^2^Department of Psychology and Behavioral Sciences, Zhejiang University, Hangzhou, China; ^3^Shanghai Institute of Technical Physics of the Chinese Academy of Sciences, Shanghai, China; ^4^University of Chinese Academy of Sciences, Beijing, China

**Keywords:** microsaccade, attention, covert attention, external attention shift, internal attention shift

## Abstract

Microsaccades are small, involuntary eye movements that occur during fixation. Since the 1950s, researchers have conducted extensive research on the role of microsaccades in visual information processing, and found that they also play an important role in human advanced visual cognitive activities. Research over the past 20 years further suggested that there is a close relationship between microsaccades and visual attention, yet lacking a timely review. The current article aims to provide a state-of-the-art review and bring microsaccades studies into the sight of attention research. We firstly introduce basic characteristics about microsaccades, then summarized the empirical evidence supporting the view that microsaccades can reflect both external (perception) and internal (working memory) attention shifts. We finally conclude and highlight three promising avenues for future research.

## 1 Introduction

Even during fixation, the human eye exhibits continuous and minute movements, including tremor, drift, and microsaccade. Among them, microsaccades are the largest and are defined as a type of involuntary small saccades that occur during fixation (Zuber et al., 1964). Over the past 70 years, researchers have conducted extensive investigations into the physiological underpinnings of microsaccades and their roles in low-level retinal visual processing (for reviews see Poletti and Rucci, 2016; Alexander and Martinez-Conde, 2019; Hafed et al., 2021). Substantial progress has also been made in elucidating the intricate relationship between microsaccades and high-level visual cognitive processes, revealing noteworthy connections between microsaccades and attention (e.g., Hafed and Clark, 2002; Engbert and Kliegl, 2003; for reviews see Rolfs, 2009; Martinez-Conde et al., 2013), working memory (e.g., Gao et al., 2015; Dalmaso et al., 2017), and consciousness (White and Rolfs, 2016). Of particular note, the association between microsaccades and attention has garnered increasing interest in recent years (e.g., Yuval-Greenberg et al., 2014; Tian et al., 2016; Lowet et al., 2018; van Ede et al., 2019; Liu et al., 2022; Yu et al., 2022), accumulating piles of empirical evidence that suggest relatively clear and consistent patterns. However, a comprehensive and up-to-date review on this topic is currently in lack, which hinders the integration of microsaccades into attention research to a certain extent. Consequently, the present study endeavors to furnish a thorough overview of the interrelated interplay between microsaccades and attention, while offering valuable insights into prospective avenues for future research.

## 2 Basic characteristics about microsaccades

**Figure 1:**
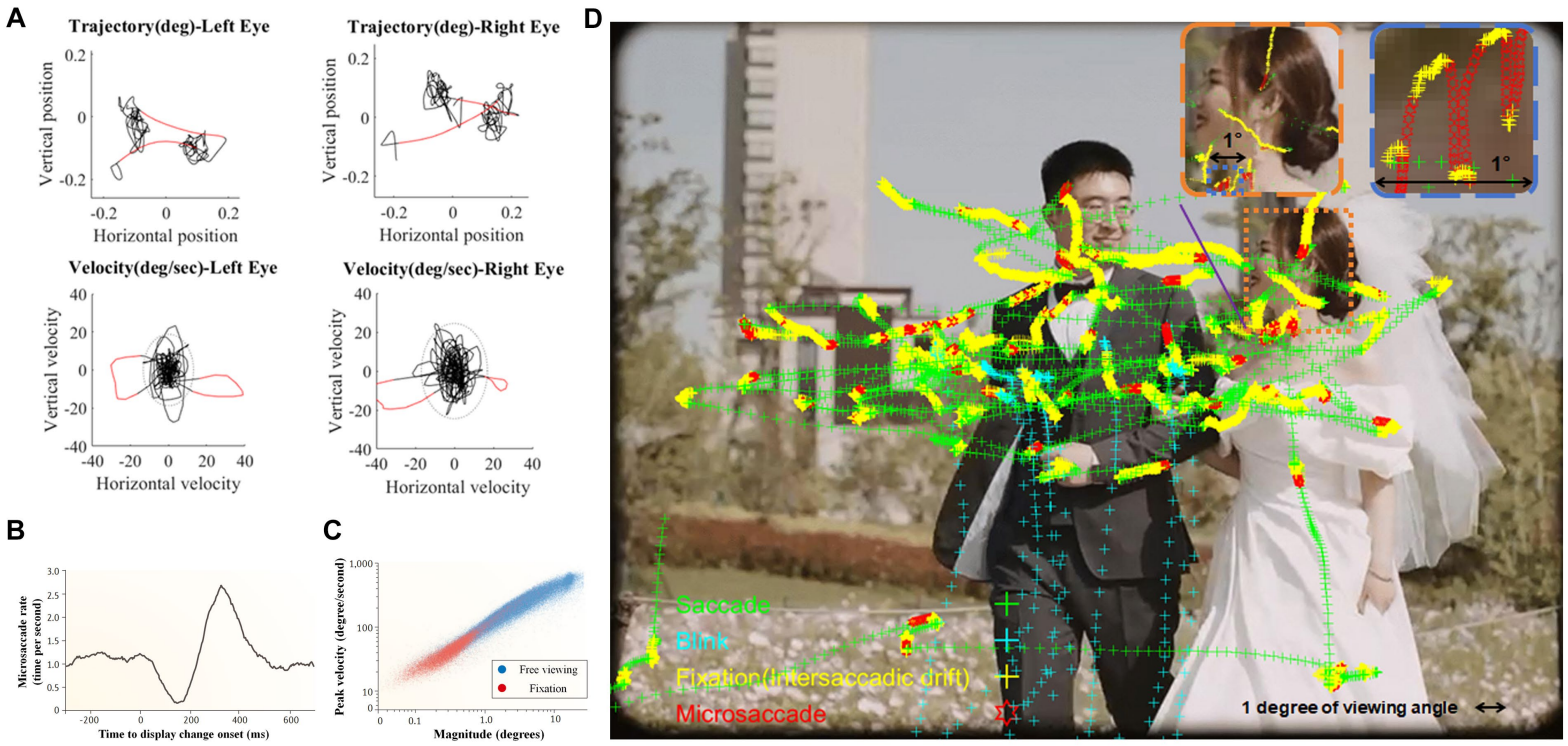
**(A)** An example of eye movement trajectories and velocities distribution of both eyes during 1-s fixation. According to relative speed, detected microsaccades are highlighted in red. **(B)** When change takes place in the attended visual display, microsaccade rate showed distinct “inhibition-rebound” characteristics (modified from Engbert and Kliegl, 2003); **(C)** Microsaccades and saccades during free viewing (blue dots) follow the same main sequence (i.e., highly correlated saccade amplitudes and peak velocities) as microsaccades produced during fixation (red dots; Martinez-Conde et al., 2013); **(D)** A demonstration of microsaccades and saccades during free viewing. Figures 1B,C are reproduced with permission from Springer Nature (i.e., one picture of the 1st author’s wedding day).

Microsaccades are characterized as ballistic eye movements, exhibiting small, linear trajectories within their overall motion path (see Figure 1A). These movements typically occur at a rate of 1–3 times per second and have durations ranging between 6 and 30 milliseconds (e.g., Alexander and Martinez-Conde, 2019). The temporal intervals between successive microsaccades align with an exponential distribution, indicative of a Poisson process, thereby underscoring their stochastic nature (Engbert, 2006). In comparison to ocular tremors and drifts, microsaccades demonstrate superior velocity (used as the most typical method to detect microsaccades, see Engbert and Kliegl, 2003), averaging between 6 and 120 degrees per second, and exhibit larger amplitudes, extending up to 1 degree of visual angle^1^. Furthermore, microsaccades are predominantly binocular in nature and exhibit a directional bias toward horizontal movements (for review see Hauperich et al., 2019).

The frequency of microsaccade is modulated both by external stimuli and internal states. Alterations in the visual field typically result in an initial suppression of microsaccade frequency, followed by a pronounced increase to a peak before a gradual return to baseline levels (e.g., Engbert and Kliegl, 2003; see Figure 1B). This phenomenon of microsaccadic suppression is influenced by the physical properties of the stimulus, such as contrast and spatial frequency (Bonneh et al., 2015) and is contingent upon the observer’s perceptual awareness (White and Rolfs, 2016). It is also associated with the phase resetting in the ongoing microsaccadic rhythmic oscillations (Hafed and Ignashchenkova, 2013). Additionally, the anticipation of stimulus presentation (e.g., Yu et al., 2016; Amit et al., 2019) and action preparation (Betta and Turatto, 2006; Watanabe et al., 2013) can lead to a prolonged inhibition of microsaccades^2^.

In recent years, research on microsaccades has expanded beyond strict fixation to include their role in the exploration of natural scenes (see Figure 1D; for reviews, see Martinez-Conde et al., 2013; Piras and Raffi, 2023). In the context of navigating through natural environments, microsaccades demonstrate a significant overlap with conventional saccades (for reviews, see Martinez-Conde et al., 2013; Krauzlis et al., 2017). This similarity extends to their underlying neurophysiological mechanisms (e.g., Hafed and Krauzlis, 2012) and adherence to the ‘main sequence’ relationship, which is characterized by a consistent linear correlation between peak saccadic velocity and amplitude (Otero-Millan et al., 2008; illustrated in Figure 1C). Given these similarities, it becomes crucial to differentiate between these two types of saccadic movements. The prevailing scholarly view emphasizes the more reflexive and less voluntary nature of microsaccades in comparison to small saccades (e.g., Mergenthaler and Engbert, 2010; Sinn and Engbert, 2016; see Willeke et al., 2019 for a different perspective).

## 3 Microsaccade direction and attention shifts

Eye movements and visual attention have long been believed to have a close relationship. In comparison to overt attention, which is accompanied by noticeable eye movements, researchers have shown particular interest in covert attention, which lacks obvious eye movements (Posner, 1980). Studies have consistently revealed that microsaccades are closely associated with covert attention shifts. The supporting evidence primarily comes from two lines: external attention that focused on visual perception, and internal attention which is directed toward intrinsic representational systems such as working memory (e.g., Chun et al., 2011; Kiyonaga and Egner, 2013).

### 3.1 Microsaccades can reflect external attention shifts

Early research investigated whether microsaccades could reflect external attention shifts. Hafed and Clark (2002) first investigated this topic, conducting experiments where participants were instructed to maintain central fixation while attending to four peripheral target locations. A sequence of visual cues presented randomly between the fixation and a peripheral target, inducing covert attention shifts. Subsequent to an unpredictable number of cues, the most recently cued target location displayed a color after a variable cue-target interval, and participants identified the displayed color. The results revealed an early microsaccades bias toward the target location (170 to 340 ms post-cue). Furthermore, this bias correlated with an enhanced target discrimination performance in trials characterized by that particular cue-target interval. A second experiment used anti-cues, wherein the cue consistently indicated a direction opposite to the actual target, and revealed that microsaccades were initially attracted to the cue side, then shifted toward the target side. Notably, in this scenario, optimal target discrimination was achieved with longer cue-target intervals. These findings provide compelling evidence for a direct relationship between the directionality of microsaccades and real-time attention shifts. Subsequent studies examined four key characteristics that link microsaccade directionality with attentional shifts: (1) Influence of attentional cues: Both endogenous and exogenous cues are known to precipitate shifts in attention. Endogenous cues, however, induce these shifts more gradually and with a subtler impact compared to exogenous cues (e.g., Müller and Rabbitt, 1989). The direction of microsaccade aligns well with such difference in attention shifts: exogenous cues caused a fast and strong microsaccade direction bias toward the cue, whereas endogenous cues only caused a late and weak bias (Engbert and Kliegl, 2003; Laubrock et al., 2005). These results suggest that microsaccade direction can distinguish different types of attention shifts, strengthening a direct correlation between attention shifts and microsaccade directionality. (2) Enhancements in early visual cortex: Meyberg et al. (2015) found that attention shifts correlate with changes in the early visual cortex, as indicated by a pronounced P1/N1 microsaccade-related potential component at occipital scalp sites. Notably, these changes are ipsilateral to the direction of microsaccades and emerge subsequent to the bias in microsaccade direction, suggesting a temporal and spatial link between microsaccade direction and neural activity associated with attention shifts. (3) Inhibition of Return (IOR) phenomenon: The IOR effect, where attention is less likely to return to a previously attended location (Posner and Cohen, 1984), also influences microsaccade direction. In scenarios involving invalid cues and the occurrence of IOR, microsaccades are more frequently oriented in the direction opposite to the cue (Galfano et al., 2004; Betta et al., 2007). This indicates that the direction of microsaccades is not only a response to immediate attentional cues but also to the overall pattern of attention shifts. (4) Microsaccade direction and attention-related performance: Evidence particularly from nonhuman primate studies underscores a direct relationship between microsaccade direction and attentional performance. Hafed et al. (2011) observed that when microsaccades were directed toward a cued location soon after a target appeared, the subjects exhibited a higher likelihood of target discrimination. Conversely, when microsaccades were oriented away from the cue, the likelihood of successful discrimination decreased. This underscores the functional significance of microsaccade direction in tasks that require attention.

Researchers further investigated whether microsaccades could directly influence or even cause attentional activity. Yuval-Greenberg et al. (2014) conducted the first study from the perspective of how microsaccades influence covert attention allocation. After real-time detecting participants’ spontaneous microsaccades during fixation, probe items were briefly presented surrounding the initial fixation (Experiment 1) or the actual eye position (Experiment 2). Participants were required to discriminate the orientation of the target from probe items according to a postcue that either pointed to the direction of the microsaccade or opposite to it. In both Experiments, they found that participants exhibited significantly better recognition performance for stimuli presented in the direction of the microsaccades compared to the opposite direction. This result revealed a direct impact of microsaccades on the allocation of visual attention, although this effect only occurred for binocular microsaccades (Zhang et al., 2016). Lowet et al. (2018) provided further neurophysiological evidence. They recorded the influence of microsaccades on attention-related modulation of neuronal activity in macaque V4 and the inferior temporal cortex, and observed enhanced neural processing occurred only following microsaccades directed toward the attended location. However, a number of empirical investigations have yielded consistently contrary findings to the aforementioned perspective. At the behavioral level, researchers found that spatial perception is altered prior to microsaccade onset (Hafed, 2013), and there is an improvement in visual discrimination at the upcoming microsaccade location as well as an impairment at the opposite location prior to microsaccade onset (Shelchkova and Poletti, 2020). At the neural level, neurons in the superior colliculus and frontal eye fields exhibit attention-related modulation prior to microsaccades onset (Chen et al., 2015), even in the absence of microsaccades (Yu et al., 2022). Given the accumulating body of evidence suggesting an earlier emergence of attention-related activities compared to microsaccade onset, the current consensus does not support the notion that microsaccades directly cause external attentional activity.

Researchers continue to broaden our understanding of how microsaccades reflect external attention shifts from various perspectives. For example, a simple model including microsaccade generation and peri-microsaccadic changes in vision sufficiently accounted for attention capture and IOR (Tian et al., 2016). Dynamic changes in microsaccade amplitudes differentiated attention shifts under different cue validities (Lv et al., 2022). Attention shifts induced by emotional stimuli modulated microsaccadic activity (Kashihara et al., 2014). In more ecologically valid environments, some studies replicated the link between attention and microsaccade direction (e.g., Barnhart et al., 2019; Xue et al., 2020), while others found that this relationship disappears with increasing environmental complexity (Willett and Mayo, 2023).

Overall, current results suggest that although microsaccades may not be deterministic for external attention activities, the direction of them still can be regarded as a useful, yet not perfect, marker of external attention shifts. Specifically, express microsaccades occurring 60–100 ms after cue presentation (Tian et al., 2018), microsaccades occurring 200–400 ms after cue presentation, the first microsaccade occurring after cue presentation (Laubrock et al., 2010), and microsaccades occurring during periods of extremely low microsaccade rate (Pastukhov and Braun, 2010) are believed to provide a more accurate reflection of attention shifts.

### 3.2 Microsaccades can reflect internal attention shifts

**Figure 2:**
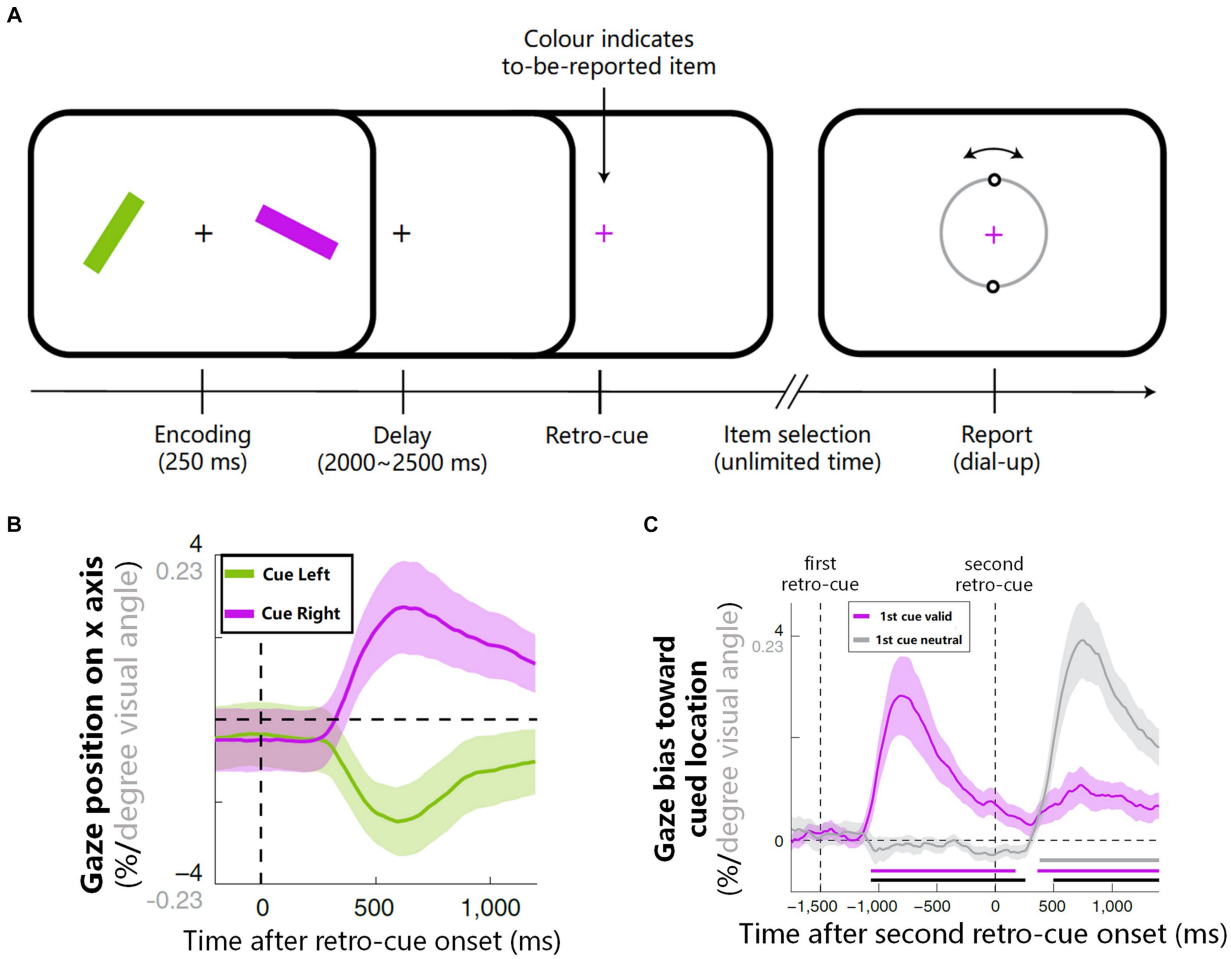
The procedure and results on how microsaccades reflect internal attention shifts (van Ede et al., 2019). **(A)** Experimental procedure: participants memorized orientations of two colored bars and then reproduced the orientation of one bar according to the color of retro-cue. **(B)** After the onset of retro-cue, the average gaze position involuntarily biased toward the memorized location of the cued item. **(C)** When there were two retro-cues (see Experiment 2 of van Ede et al., 2019), if the first retro-cue was neutral, gaze shift did not emerge until the valid retro-cue onset (gray line); if the first retro-cue was valid, only the onset of the first cue led to a strong involuntary gaze shift (purple line). Figures 2A–C are reproduced with permission from Springer Nature.

In recent years, there has been growing interest in using eyetracking to reflect internal attention shifts. van Ede et al. (2019) conducted a pioneering study investigating the relationship between gaze shifts and attention shifts in working memory. They required participants firstly to memorize the orientations of two colored bars and then to reproduce the orientation of one bar according to the color of the fixation (see Figure 2A), while analyzing the precise gaze position during the maintenance period. The results revealed that after the onset of colored retro-cue and it guided attention to a particular representation in working memory, the gaze position involuntarily shifted, in line with microsaccades’ magnitudes, slightly toward the location where that representation was once presented (see Figure 2B). Moreover, when there were two retro-cues and the first can be either valid or neutral (see Experiment 2 in van Ede et al., 2019), the pattern of gaze shifts did not emerge until the valid retro-cue presented (see Figure 2C). These findings support the notion that microsaccades can serve as an indicator of internal attention shifts, rather than sustained attention biases. Follow-up research further demonstrated that such reflection owned a high temporal precision (van Ede et al., 2021), and could be achieved via both voluntary and involuntary attention selection (van Ede et al., 2020).

Similar to research on external attention shifts, researchers also examined whether microsaccades can directly influence or cause internal attention shifts. Behavioral studies suggested that microsaccades do not cause internal attention shifts, since that an involuntary gaze-shift manipulation did not bring an item into the focus of attention so as to benefit subsequent performance (van Ede et al., 2019). This perspective is further corroborated by neural research, notably by Liu et al. (2022). They used lateralized alpha EEG as an objective reference for internal attention (see van Ede, 2018 for a review) and revealed a close relationship between microsaccade direction and alpha lateralization: alpha lateralization is stronger in trials with microsaccades toward versus away from the memorized location, and occurs earlier when microsaccades orient to the target earlier. Intriguingly, alpha lateralization was still observable even in the absence of microsaccades, supporting that internal attention shifts can occur independently of microsaccades. However, Liu et al. (2023) also noted that microsaccades can elicit stronger alpha activity when they oriented toward a specific direction, facilitating transient lateralized alpha activity. It implied that microsaccades may modulate internal attention to some extent. Given these insights, it is imperative for future research to further explore the influence of microsaccades on internal attention processes and to elucidate the underlying mechanisms through which microsaccades reflect shifts in internal attention.

## 4 Conclusion and future directions

In summary, current results suggest that the directionality of microsaccades can serve as a valid, yet not perfect, indicator of both external and internal attention shifts. Meanwhile, bunch of studies do not support the notion that microsaccades directly cause attention shifts. To achieve a comprehensive understanding of the interplay between microsaccades and attention, it is essential to introduce the study of microsaccades into various other attention-related cognitive domains. In this vein, we propose three prospective trajectories for future research:

1. The potential of microsaccades as indicators of non-spatial attentional engagement is a subject of considerable interest in the field of cognitive neuroscience. Currently, research on whether and how microsaccades reflect attentional activities mainly focuses on spatial attention, with few studies tapping on temporal attention (e.g., Denison et al., 2019; Palmieri et al., 2023) and other modalities beyond visual scope (e.g., auditory attention, Contadini-Wright et al., 2023; tactile perception, Badde et al., 2020). Notably, even within the realm of visual attention, the exploration of several critical attentional activities remains largely underrepresented, including feature-based attention (Maunsell and Treue, 2006), object-based attention (Chen, 2012; Baldauf and Desimone, 2014), and social attention (Birmingham and Kingstone, 2009; Nummenmaa and Calder, 2009). Future research trajectories would benefit greatly from a more holistic examination of the role of microsaccades across these varied types of attentional activities. Such an approach would be instrumental in unraveling the intricate mechanisms that underpin the interplay between microsaccades and attentional processes, thereby enriching our comprehension of both microsaccades related cognitive functions and different types of attention activities.
2. The mechanisms by which microsaccades reflect shifts in internal attention are at the forefront of contemporary cognitive research, with significant advancements noted in recent literature (Griffin and Nobre, 2003; Souza and Oberauer, 2016; van Ede and Nobre, 2023). This reflection is potentially attributable to the involuntary encoding of location information with memory representations (e.g., Treisman and Zhang, 2006; Gu et al., 2020), coupled with an active rehearsal of memory representations based on their relative positions in the focus of attention (de Vries and van Ede, 2024). One intriguing aspect for future examination is the dependency of microsaccade directionality on the spatial arrangement of information at the time of memory encoding, the relative positions among memory representation, as well as the interaction between the two factors. Expanding this line of inquiry is anticipated to significantly enhance our comprehension of internal selective attention mechanisms. Furthermore, it proposes to extend the exploration of microsaccadic reflections beyond controlled experimental settings (Draschkow et al., 2022), potentially leading to insights applicable in real-world scenarios.
3. The application of microsaccade in reflecting attention shifts. Microsaccades offer notable advantages over traditional attention shift markers: they occur more rapidly (e.g., express microsaccades occurring within 100 ms, see Tian et al., 2018), are less influenced by subjective will, and are particularly relevant in tasks requiring fixation without overt saccades. Unlike the N2pc (N2-posterior-contralateral) component, a commonly used event-related potential in EEG studies for tracking attention shifts, microsaccades are not constrained by specific time windows or rigorous experimental conditions. Thus, microsaccades have great potential as a valuable tool in cognitive neuroscience, enhancing our understanding of complex attentional mechanisms. For example, in the field of visual perception, microsaccades can help researchers study how the brain ignores salient yet distracting stimuli (for reviews see Gaspelin and Luck, 2018; Theeuwes, 2023). This involves examining whether microsaccades are drawn to distractions before participants make any noticeable eye movements (e.g., Gaspelin et al., 2017). In the realm of working memory, microsaccades can reveal the nuances of attentional guidance (van Loon et al., 2017), serving as a finer detector for revealing attentional guidance process, thereby enhancing our comprehension of cognitive processes underpinning attention and working memory.

## Ethics statement

Written informed consent was obtained from the individual(s) for the publication of any identifiable images or data included in this article.

## Author contributions

QG: Conceptualization, Funding acquisition, Investigation, Methodology, Project administration, Resources, Software, Visualization, Writing – original draft, Writing – review & editing. QZ: Investigation, Writing – original draft. YH: Investigation, Methodology, Writing – original draft. PL: Methodology, Visualization, Writing – original draft. ZG: Conceptualization, Resources, Supervision, Writing – review & editing. MS: Conceptualization, Resources, Supervision, Writing – review & editing.

## Footnotes

^1^There is currently no consensus on the precise range for defining the amplitude of microsaccades. As eye tracking technologies evolve and experimental contexts vary over time, the standards continue to evolve as well [see Rolfs (2009) for a comprehensive review]. Researchers commonly employ a criterion of a maximum of 1 to 2 degrees of visual angle.

^2^The perceptual functions of microsaccade and the neural bases of its generation are beyond the scope of current study. For extensive reviews see Alexander and Martinez-Conde (2019); Rucci and Poletti (2015); Poletti and Rucci (2016); Krauzlis et al. (2017).
